# Bilateral vestibulopathy: beyond imbalance and oscillopsia

**DOI:** 10.1007/s00415-020-10243-5

**Published:** 2020-10-08

**Authors:** F. M. P. Lucieer, R. Van Hecke, L. van Stiphout, S. Duijn, A. Perez-Fornos, N. Guinand, V. Van Rompaey, H. Kingma, M. Joore, R. van de Berg

**Affiliations:** 1 Department of Otorhinolaryngology and Head and Neck Surgery, Division of Balance Disorders, School for Mental Health and Neuroscience, Maastricht University Medical Center, Maastricht, The Netherlands; 2 Department of Rehabilitation Sciences, University of Ghent, Ghent, Belgium; 3 Faculty of Health, Medicine and life Sciences, University of Maastricht, Maastricht, The Netherlands; 4 Service of Otorhinolaryngology Head and Neck Surgery, Department of Clinical Neurosciences,, Geneva University Hospitals, Geneva, Switzerland; 5 Faculty of Physics, Tomsk State Research University, Tomsk, Russian Federation; 6 Department of Otorhinolaryngology and Head and Neck Surgery, Antwerp University Hospital, Faculty of Medicine and Health Sciences, University of Antwerp, Antwerp, Belgium; 7 Department of Clinical Epidemiology and Medical Technology Assessment (KEMTA), Care and Public Health Research Institute (CAPHRI) of the Faculty of Health, Medicine and Life Sciences of Maastricht University (FHML), Maastricht University Medical Centre, Maastricht, The Netherlands

**Keywords:** Bilateral vestibulopathy, Bilateral vestibular hypofunction, Outcome measures, Symptoms, Vestibular implant, Vestibular prosthesis

## Abstract

**Objective:**

To optimize the current diagnostic and treatment procedures for patients with bilateral vestibulopathy (BV), this study aimed to determine the complete spectrum of symptoms associated with BV.

**Method:**

A prospective mixed-method study design was used. Qualitative data were collected by performing semi-structured interviews about symptoms, context, and behavior. The interviews were recorded and transcribed until no new information was obtained. Transcriptions were analyzed in consensus by two independent researchers. In comparison to the qualitative results, quantitative data were collected using the Dizziness Handicap Inventory (DHI), Hospital Anxiety and Depression Scale (HADS) and a health-related quality of life questionnaire (EQ-5D-5L).

**Results:**

Eighteen interviews were transcribed. Reported symptoms were divided into fourteen physical symptoms, four cognitive symptoms, and six emotions. Symptoms increased in many situations, such as darkness (100%), uneven ground (61%), cycling (94%) or driving a car (56%). These symptoms associated with BV often resulted in behavioral changes: activities were performed more slowly, with greater attention, or were avoided. The DHI showed a mean score of severe handicap (54.67). The HADS questionnaire showed on average normal results (anxiety = 7.67, depression = 6.22). The EQ-5D-5L demonstrated a mean index value of 0.680, which is lower compared to the Dutch age-adjusted reference 0.839 (60–70 years).

**Conclusion:**

BV frequently leads to physical, cognitive, and emotional complaints, which often results in a diminished quality of life. Importantly, this wide range of symptoms is currently underrated in literature and should be taken into consideration during the development of candidacy criteria and/or outcome measures for therapeutic interventions such as the vestibular implant.

**Electronic supplementary material:**

The online version of this article (10.1007/s00415-020-10243-5) contains supplementary material, which is available to authorized users.

## Introduction

Bilateral vestibulopathy (BV) is a chronic vestibular disorder in which the vestibular function is bilaterally absent or severely reduced [1, 2]. Currently, the reported prevalence of BV in literature varies between 28 and 81 in 100.000 adults. This is probably an underestimation of the accurate prevalence, since BV is often missed or misdiagnosed [3–5]. BV is mainly diagnosed using the criteria reported by the Barany Society, which include a combination of symptoms and vestibular test results. The symptoms mentioned in the criteria include imbalance, oscillopsia, and worsening of complaints in darkness and/or on uneven ground [6], which lead to an increased risk of falling [7]. However, these diagnostic criteria do not intend to cover the whole spectrum of symptoms and consequences associated with BV. This should be acknowledged, since clinical experience and earlier performed retrospective studies showed a wide variety of symptoms and consequences [8]. For example, BV patients reported negative impact on physical and social functioning, and compromised cognitive abilities as well [3, 4, 8–13]. Therefore, it is important to investigate this whole spectrum of symptoms and consequences, especially when developing candidacy criteria and/or outcome measures for therapeutic interventions in this patient population [14].

Currently, an effective treatment that restores peripheral vestibular function does not exist in clinical practice [3, 4, 15, 16]. Vestibular rehabilitation is currently a recommended treatment option which could improve gaze stability, gait and static postural instability [17]. However, it does not restore the function. Research on galvanic vestibular stimulation which uses surface electrodes shows some promising results regarding improvement of balance and gait [18–20]. The vestibular implant is another possible treatment option for the future. The feasibility of a vestibular implant has previously been demonstrated, including (partial) restoration of the vestibulo-ocular reflex and vestibulo-collic reflex, elicitation of controlled postural responses and proof of the first functional benefits (normalization of dynamic visual acuity) [21–25]. However, the benefit of the vestibular implant on quality of life of BV patients still needs to be demonstrated.

Questionnaires currently used to evaluate the impact of vestibular deficits on quality of life, like the Dizziness Handicap Inventory (DHI), are not specifically developed for BV [26]. This might impede thorough evaluation of BV patients before and after therapeutic interventions, like the vestibular implant. Previous to this study, a systematic review was performed which illustrated that a few clinical studies and case reports mentioned additional symptoms next to the classic symptoms (imbalance and oscillopsia). However, none of these provided a comprehensive overview of the spectrum of symptoms related to BV [8]. Therefore, the objective of this study was to determine the complete spectrum of BV symptoms and its consequences by performing a prospective mixed-method study. This could facilitate the development of candidacy criteria for therapeutic interventions and a specific patient-reported outcome measure (PROM) for BV [14, 27].

## Methods

### Inclusion criteria

Fifty patients diagnosed with BV according to the Barany criteria were included [6]. Accordingly, all patients reported imbalance and/or oscillopsia during walking or head movements, and had a reduced bithermal caloric response (sum of bithermal mean peak slow phase eye velocity on each side < 6°/s) and/or a reduced vestibular–ocular reflex (VOR) gain [< 0.6 bilaterally measured by the horizontal video head impulse test (VHIT), and/or reduced horizontal angular VOR gain < 0.1 measured by a sinusoidal stimulation on a rotatory chair (0.1 Hz)] [6]. Subjects who were not able (e.g., mentally disabled) or willing to talk about one of the investigated topics (e.g., psychology/psychiatry, health care utilization), were not able to stop medication against anxiety or depression (due to the vestibulo-suppressive effect), or willing to undergo one of the detailed physical, audiometric or vestibular examinations were excluded from participation in this study. All subjects were interviewed and underwent vestibular testing at Maastricht University Medical Center + by the same examiner (FL).

### Vestibular testing

According to the Barany criteria, three vestibular assessments were performed on the day of the interview, to confirm the diagnosis of BV. The caloric test was performed in a completely dark room. In a supine position, warm (44 °C) and cold (30 °C) water irrigations of at least 250 milliliters were administered for 30 s. Eye movements were recorded with electronystagmography (KingsLab 1.8.1, Maastricht University, Maastricht, the Netherlands).

The video head impulse test was performed using the Otometrics system (Otometrics, Taastrup, Denmark). The testing method was previously described [28]. In summary, the patient was sitting in an immobile chair and was instructed to look at a fixed target on the wall at 1.5 m. Unpredictable head impulses with a velocity of > 150°/s and low amplitude (± 20°) were applied in the plane of both horizontal semicircular canals. At least seven impulses were applied in each direction.

In a completely dark room, the torsion swing test was performed using a rotatory chair (Ekida GmbH, Buggingen, Germany) that sinusoidally rotated at a frequency of 0.1 Hz and a peak velocity of 60°/s. Eye movements were recorded with electronystagmography (KingsLab 1.8.1, Maastricht University, Maastricht, The Netherlands).

### Qualitative research analysis: interviews

A semi-structured interview was completed with each patient about symptoms, emotions, context, and behavior related to BV. The BV patients were asked open-ended questions about what kind of symptoms they experienced (somatic and psychological); which symptom was most frequently present; which symptoms were most disturbing in daily life; in which situations (outside and inside their house) these symptoms were noticed; how they were dealing with these symptoms (behavior and emotions); and the influence of the disease on their relationships. In case a patient did not know what to answer, examples obtained from clinical experience or previous interviews were given. All interviews were recorded. Verbatim transcription of the number of interviews was continued until no new information was obtained [29, 30]. The first and second authors (FL and RVH) independently assigned open codes through isolation of words or statements, for all transcribed interviews. The list of codes per interview was discussed. In consensus, all retrieved complaints were divided into main categories. Subsequently, the two investigators independently coded and formulated subcategories of symptom descriptions. These subcategories were labeled. In addition, the emotional complaints were separately categorized according to Parrot’s classification of emotions into primary, secondary, and tertiary emotions (complete classification: see supplementary materials). The primary emotions are love, joy, surprise, anger, sadness, and fear [31]. In case of a disagreement between the two independent researchers, the original data were reassessed together to reach consensus. Mind maps were created using Mindomo (version 9.2.4).

### Quantitative research analysis: questionnaires

The patients received three symptom-related questionnaires at least two weeks before the interview: The Dizziness Handicap Inventory (DHI), the Hospital Anxiety and Depression Scale (HADS) and the EuroQol-5D-5L (EQ-5D-5L). They were instructed to complete the three questionnaires at home one day before the interview or on the day of the interview. These questionnaires were chosen to get an insight into their vestibular complaints, mood, and overall health status. The DHI is an instrument which is commonly used in patients with vestibular complaints. It quantifies the impact of dizziness on daily life by measuring three dimensions: physical (max. 28 points), functional (max. 36 points), and emotional (max. 36 point). The total score (range 0–100) of these subscales provides information about the experienced handicap (< 16, no handicap; 16–34 mild handicap; 36–52 moderate handicap; ≥ 54 severe handicap) [4, 32]. The HADS questionnaire indicates present anxiety and depression levels of a patient. Anxiety and depression subscores of 8–10 are considered borderline and scores above 10 are considered pathological [33, 34]. The EQ-5D-5L is used for measuring generic health status. It comprises five dimensions (mobility, self-care, usual activities, pain/discomfort, and anxiety/depression) and a visual analog scale. Outcomes can be compared to Dutch age-adjusted references [35–37].

### Mixed method: comparing qualitative and quantitative research results

After the qualitative and quantitative data were analyzed individually, a mixed-method approach was used. This methodology of research provided the opportunity to integrate a variety of perspectives and combines the strength of both qualitative and quantitative data. A concurrent triangulation approach was chosen to merge and compare these results [38–41]. The quantitative and qualitative data were prioritized equally. A joint display was created providing a cognitive framework for integration of the collected data [40, 42]. For each discrepancy between quantitative and qualitative outcomes, a relevant quotation per patient was identified.

### Ethical considerations

This study was conducted in accordance with the legislation and ethical standards on human experimentation in the Netherlands and in accordance with the Declaration of Helsinki (amended version 2013). Approval was obtained from the ethical committee of Maastricht University Medical Center (NL52768.068.15/METC). All procedures were performed at the Maastricht University Medical Center. All subjects provided written informed consent.

## Results

### Patient characteristics

Eighteen BV patients [mean age 60 years (range 21–71 years), 11♀] were included for the qualitative analysis. The etiologies of BV in these patients were: ototoxicity (gentamicin *n* = 3; amikacin *n* = 1); Hashimoto’s disease (*n* = 1); renal failure (*n* = 1); vestibular neuritis (*n* = 1); bilateral Ménière’s disease (*n* = 2); congenital (*n* = 1); and idiopathic (*n* = 8, of which 4 reported a migraine history).

Sixteen (89%) BV patients had a bilaterally reduced caloric response, 12 (67%) had a bilaterally reduced VOR gain as measured with VHIT, and ten (56%) showed a reduced VOR gain on the torsion swing test. Eight (44%) BV patients met all three diagnostic criteria on vestibular function [6].

Fifty BV patients [mean age 60 years (range 21–79 years), 25♀], including the above-mentioned eighteen BV patients, completed the questionnaires. The etiologies were ototoxicity (*n* = 11), infection (neuritis *n* = 1, meningitis *n* = 3, Lyme’s disease *n* = 1, herpes infection *n* = 1), hereditary (DFNA9 gene mutation *n* = 3, other *n* = 2), congenital *n* = 1, Menière’s disease (*n* = 3), auto-immune (*n* = 2), renal failure (*n* = 1), and idiopathic (*n* = 21, of which 9 reported a migraine history). Forty-five BV patients (90%) had a bilaterally reduced caloric response, 39 patients (78%) had a bilaterally VOR gain < 0.6 measured with the video head impulse test, and 30 patients (60%) had a VOR gain of < 0.1 on the torsion swing test. Twenty-six BV patients (52%) met all three of the vestibular testing inclusion criteria [6].

### Qualitative results from the semi-structured interviews

After eighteen interviews, no new information was obtained and transcription was stopped. The average duration of the first eighteen interviews was 60 min (range 26–96 min) and 7235 words (range 3159– 16,940 words). The average interview duration of the total group of 50 patients was 58 min (range 26–96 min). Three main categories were identified: symptoms (physical, cognitive, emotions), context, and behavior.

#### Symptoms

**Fig. 1:**
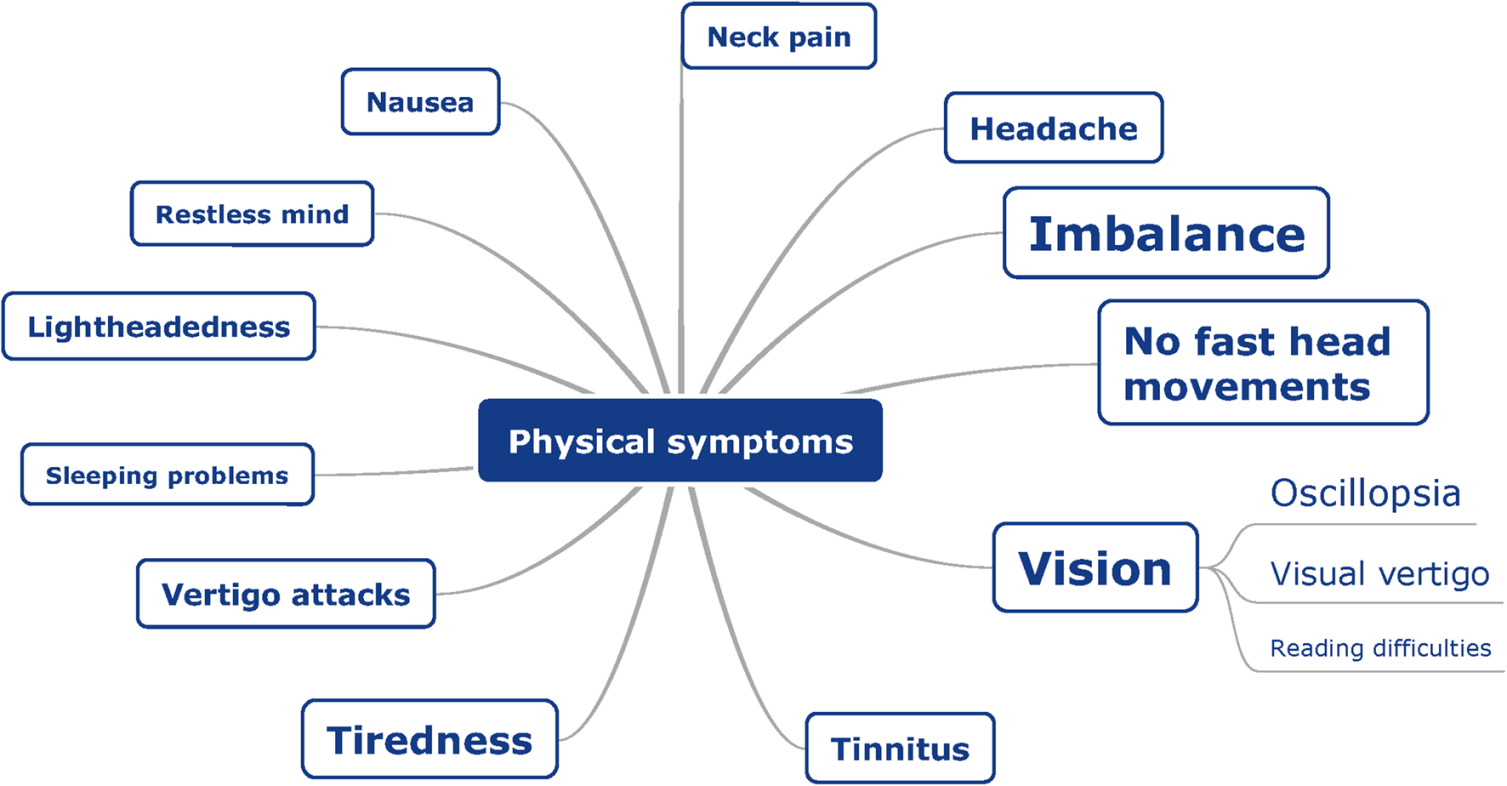
Mind map of physical symptoms, as reported by BV patients (*n* = 18) during semi-structured interviews. The larger the font size of a specific symptom, the more often this symptom was addressed during the interviews

**Fig. 2:**
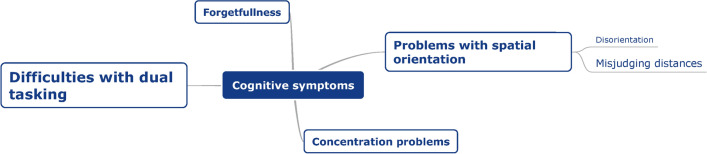
Mind map of cognitive symptoms, as reported by BV patients (*n* = 18) during semi-structured interviews. The larger the font size of a specific symptom, the more often this symptom was addressed during the interviews

**Fig. 3:**
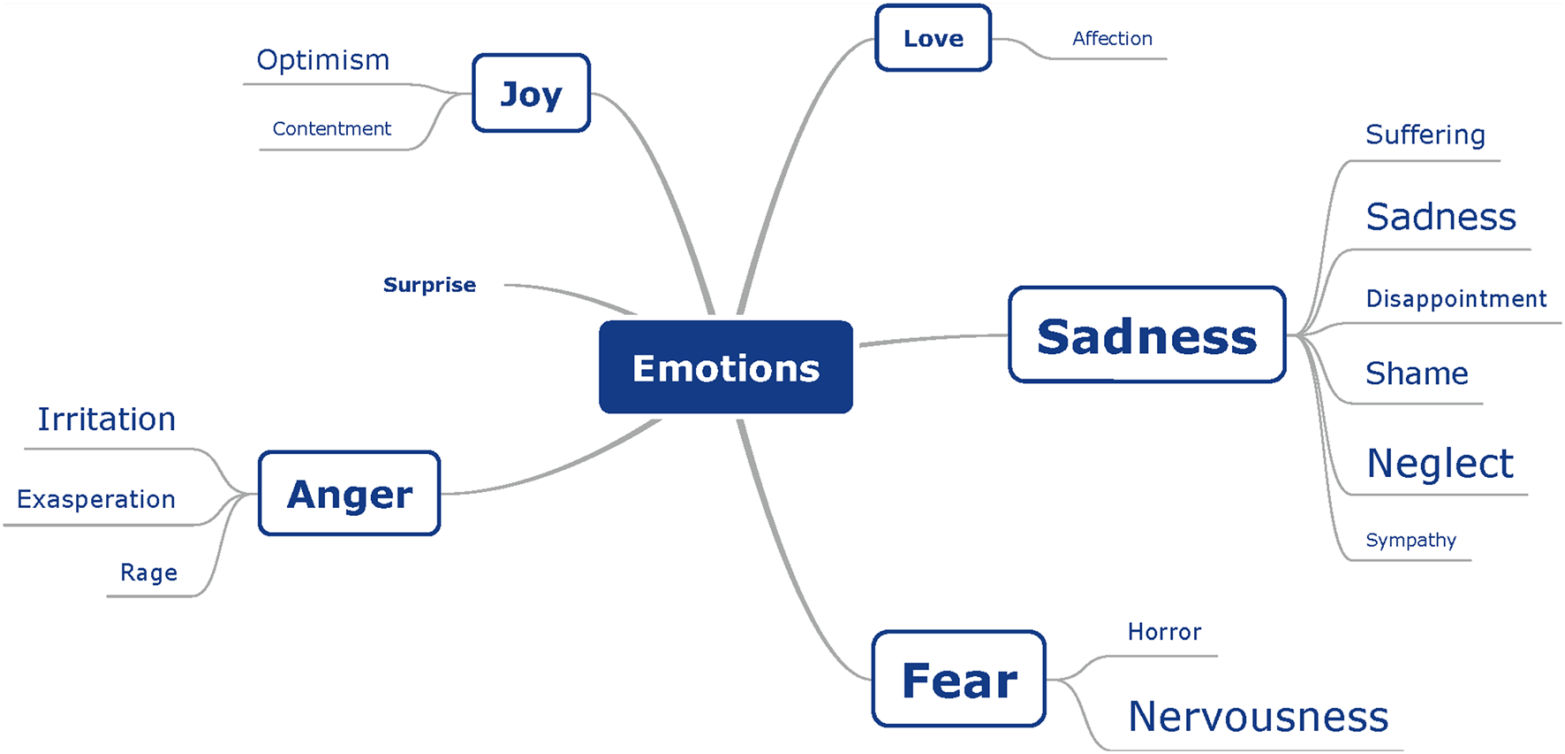
Mind map of emotions, as reported by BV patients (*n* = 18) during semi-structured interviews. The larger the font size of a specific emotion, the more often this emotion was addressed during the interviews

After coding the interviews, symptoms were divided into three main categories: physical symptoms, cognitive symptoms, and emotions. Within these large categories, the symptoms were sorted into subcategories and labeled. Figures 1, 2 and 3 present mind maps of the three categories of symptoms. In addition, the frequency of occurrence can be found in the supplementary materials.

##### *Physical symptoms (Fig. **1**)*

Imbalance was reported by all subjects. Patients reported unsteadiness during walking or standing, unsteadiness during other movements, falling, losing balance during fast movements, losing balance while cycling, and need of support or reference.

The second most common physical symptom was visual problems (94%), of which mainly oscillopsia was mentioned. Patients described a moving horizon or environment during movements, problems with recognizing faces or reading signs during walking, and visual trailing. Additionally, 14 patients (78%) mentioned visual vertigo, of which looking at fast-moving objects like a train or car, sun shining through trees, headlights of cars, and certain patterns or colors were frequently reported triggers. Eight patients (44%) mentioned reading difficulties, for example reading subtitles while watching television.

Twelve patients (67%) mentioned not being able to perform fast head movements during sitting, standing, walking, and/or cycling, as a result of loss of balance control and visual acuity. Moreover, the subjects reported tiredness (67%), tinnitus (39%), headache (39%), vertigo attacks (33%), lightheadedness (28%), neck pain (22%), nausea (22%), restless mind (17%), and sleeping problems (11%).

All subjects who complained of headaches (39%) also reported migraines in their medical history. The etiologies of these patients were neuritis (*n* = 1), bilateral Menière’s disease (*n* = 2), congenital (*n* = 1), and idiopathic (*n* = 3). The etiologies of the subjects with nausea (22%) were neuritis (*n* = 1), Menière’s disease (*n* = 2), and idiopathic (*n* = 1). Patients who reported tinnitus had different etiologies (Menière’s disease, gentamicin, idiopathic) and their hearing varied between normal and mild hearing loss with pure tone averages of 5 dB HL up till 47 dB HL (mean 25 dB HL).

##### *Cognitive symptoms *(Fig. 2)

Fifteen patients (83%) reported having problems with dual tasking. Examples which were given involved crossing the street while turning their head, cycling and crossing the street, walking while reading on their smartphone, and more generally, doing two things at the same time. For example: “Not being able to combine driving a car, reading signs, and using the navigation system” [P12].

Problems with spatial orientation were described by ten patients (56%). This was divided into two groups: disorientation problems (*n* = 5) and misjudging distances (*n* = 8). A quotation from a patient reporting problems of disorientation: “you can send me around the corner and then I don’t know where I am. I don't have that orientation” [P12]. Examples of misjudging distances were: not being able to estimate the depth of staircases; not being able to estimate the speed of another car; and difficulty estimating the distance between themselves and oncoming traffic. Seven patients (39%) also suffered from concentration problems and six (33%) reported to be (somewhat) more forgetful.

##### *Emotions *(Fig. 3)

Parrot’s classification of emotions was used to categorize the emotions into primary, secondary, and tertiary emotions [31]. Sadness and fear were the most common primary emotions (83%).

Secondary sadness symptoms were suffering (“it just controls my whole life” [P14]), sadness, disappointment, shame, neglect, and sympathy. Tertiary emotions of sadness were depression (“feeling down” [P11], “burnout” [P8], “intensely miserable” [P1]), despair, hopelessness, gloom, sadness (sadness, more emotional, crying), and unhappiness. Shame was divided into guilt and shame. Neglect was reported as alienation (“negated” [P1], “It bothers me the most, I am no longer myself” [P10]), isolation (“you are quite limited and you always have to bother someone. And you are quite dependent” [P15], “I am afraid to go out by myself” [P10]), neglect (“appearance is less important” [P16]), loneliness, rejection (“I have trouble with that. A lot of people fail to understand my situation” [P2]), defeat (“vulnerable” [P1]), insecurity, and embarrassment. One patient mentioned pity.

Fifteen patients (83%) experienced fear. Within this category, three patients mentioned panic: “panic attack” [P9] (when driving a car), “in a shopping center, I get scared and I panic” [P10]. In the category nervousness, anxiety (“I am afraid to fall and therefore, you always pay attention to the ground” [P16], anxiety to fall (during cycling) [P1,2,5,6,7,8,10,14,16,18]), tenseness (“stress” [P6,7], “nervous” [P6], “hyperventilation” [P7]), worry (“unsafe” [P1]), and uneasiness (“I feel embarrassed” [P8]) were noted. Fifty percent reported anger symptoms with irritation, exasperation (“powerless” [P1], “frustrating” [P9]), and rage.

In contrast, seven patients (39%) mentioned positive emotions of love and joy. Love (fondness) was described by one patient due to more appreciation of his son in law (“the quality of my son-in-law that I got to know … he was committed to it, he came to me to …, to take a walk with those crutches. Yes well, I appreciate that very much. Only positive in that respect … always helpful” [P18]). Joy was divided into optimism and contentment. Hope was described as “I hope to return to work” [P11] and hope for a treatment. Optimism was reported with examples of “I am quite optimistic by nature … That’s just the way it is” [P4] and “we try to make the best of it” [P11].

#### Context: challenging situations and triggers

Patients described several challenging situations in daily life. Especially situations involving darkness (100%) and uneven ground (61%) worsened their symptoms.

Seventeen patients (94%) reported multiple difficulties during cycling (unsteadiness/sway, imbalance, falling, fast head movements, tiredness, difficulties with dual tasking, misjudging distances, disorientation, fear to fall, avoidance behavior). Driving a car was difficult for ten patients (56%) due to visual vertigo, oscillopsia, not being able to turn their heads (only using the mirrors), and worsening of symptoms in darkness and on uneven ground (speed bumps). Five patients (28%) mentioned difficulties in the supermarket due to the busy environment, noisy environment, fluorescent lighting, narrow aisles, and the variety of colors. During swimming, seven patients (33%) reported difficulty perceiving the direction of gravity (e.g., knowing how to swim to the surface or bottom: “I also find it very scary when I go underwater, I just don't know which is the surface and which the bottom” [P5]), and suffered from imbalance (e.g., “I was not able to swim in a straight line and went underwater” (unintentionally) [P7], “I no longer knew where I was standing … I couldn’t keep my balance” [P11]). Regarding reading abilities, reading subtitles on television was more difficult, and some patients were not able to read for a lengthy time anymore (both 28%). Five patients (28%) reported difficulties during social activities, since they were not able to travel alone in the dark anymore. Nine patients (50%) noted they had difficulties in a busy environment in general, for example in a supermarket, shopping center, party, fair, etc. These patients mentioned complaints like anxiety, panic or feeling tense.

#### Consequences and behavior

**Fig. 4:**
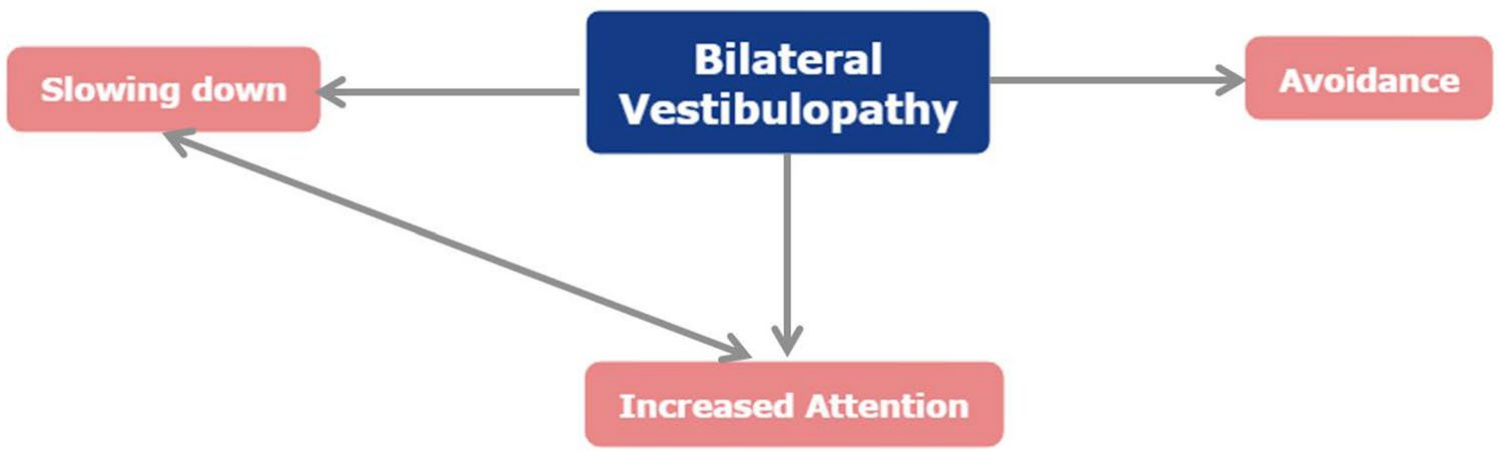
Conceptual model of altered behavior when performing specific activities and their interaction, as reported by BV patients (*n* = 18) during semi-structured interviews. Arrows indicate an interaction between items

In general, the reported symptoms led to three alterations in behavior regarding activities: activities needed to be performed more slowly, with increased attention, or were avoided. Fifteen participants (83%) reported that in order to perform activities, more concentration is needed (loss of automatism) and more energy is consumed. Consequently, more time is needed to perform daily activities, and energy is divided more throughout the day. Fourteen patients (78%) mentioned they need to do everything more slowly, at their “own pace” [P12]. Eventually, all these complaints resulted in adjustment of behavior, including avoidance behavior. Situations that were avoided, included for example: climbing ladders/stairs, going outside when it is dark, walking in the forest, swimming, driving a car, cycling, going to the supermarket on a Saturday, going to a party, etc. A conceptual model of these alterations in behavior and their interaction is presented in Fig. 4.

Patients also reported using devices to help with their complaints. Three patients use a cane and three patients use a walker outside. Four patients had installed more lights or light sensors in their houses. Two indicated using sunglasses more often, and two patients moved to a home without stairs.

BV also influenced relationships. Six patients (33%) felt misunderstood by people around them. Five patients (28%) reported loss of friendships because of BV, mainly since they were not able to visit their friends anymore in the dark, or they were not able to drive a car. Quotation of a patient: “In bad times, you get to know who your friends are” [P17]. In contrast, eight patients (44%) mentioned close relationships, like romantic relationships, and children were more worried and became more helpful.

### Quantitative results from the DHI, HADS and EQ-5D-5L

Table 1 presents the results of the DHI, HADS and EQ-5D-5L instruments obtained in 50 BV patients. In this population, the DHI showed a mean score of 56.04, indicating a severe handicap. The emotional subscale revealed the lowest score (15.72 out of max. 36 points). The HADS showed on average normal scores regarding anxiety and depression (anxiety = 6.52, depression = 6.36). The EQ-5D-5L demonstrated a mean index value of 0.680, which is lower compared to the Dutch age-adjusted reference 0.839 (60–70 years) [37]. From the five dimensions, “mobility” and “usual activities” were compromised most severely (mean score 3 out of 5, for both the 18 BV patients as well as the total group of BV patients).

**Table 1:** Results from the DHI, HADS and EQ-5D-5L obtained in 50 BV patients

Questionnaire	Mean (median, range)	
	First 18 patients	Total group (*n* = 50)
DHI		
Physical	18.33 (20, 0–26)	18.20 (20, 0–28)
Functional	20.78 (22, 6–230)	22.12 (22, 6–30)
Emotional	15.56 (16, 4–28)	15.72 (16, 4–34)
Total	54.67 (58, 12–80)	56.04 (57, 12–98)
HADS		
Anxiety	7.67 (7.0, 0–16)	6.52 (6.0, 0–16)
Depression	6.22 (4.5, 1–15)	6.36 (5.0, 0–15)
EQ-5D-5L		
VAS	67.11 (72.50, 0–85)	65.78 (70, 0–100)
Index value	0.680 (0.715, 0.439–0.837)	0.680 (0.704, 0.322–1.000)

The column “First 18 patients” refers to the 18 BV patients who underwent the qualitative analysis using the semi-structured interviews

Reference values of questionnaires:

DHI: subscales: physical (max 28), functional (max 36), and emotional (max 36). Total score (range 0–100): ≤ 16 no handicap, 16–34 mild handicap, 36–52 moderate handicap, ≥ 54 severe handicap [4, 32]

HADS: 0–7 normal, 8–10 borderline, 11–21 abnormal [33, 34]

EQ-5D-5L: VAS = visual analog scale (0—100%), index value = calculated (0–1.0). Mean index value score for age 50–60 years 0.857 and 60–70 years: 0.839 [35–37]

#### Triangulation

Table 2 presents the quantitative and qualitative results of the 18 BV patients who were analyzed both quantitatively and qualitatively. Discrepancies were found between results obtained with both analyses. Regarding the DHI, the mean DHI score indicated a severe handicap on group level, although on individual level seven patients were identified who scored “no handicap” to “moderate handicap”, while the qualitative analyses pointed to a severe disease burden. Three of those patients (patient 4, 5, 8) demonstrated lower DHI scores due to a lower score on the emotional subscale, while four patients (patients 6, 7, 13, 18) presented with lower scores on ≥ 2 subscales of the DHI. Regarding the HADS, mean scores were within the normal range, although qualitative data often indicated a higher psychological burden. For example, patient 7 scored low on the HADS subscales (anxiety 4 points, depression 3 points), but he explicitly mentioned “moments of feeling down”, “It’s all my fault”, “fear”, fear of falling, fear of walking stairs, “hyperventilating”, and “stress”. The EQ-5D-5L illustrated a reduced quality of life in all 18 BV patients, compared to their individually Dutch age-adjusted reference [37]. This corresponded with the results obtained during the semi-structured interviews.

**Table 2:** Comparison of quantitative and qualitative results of the 18 BV patients who were analyzed both qualitatively and quantitatively

	Quantitative	Qualitative			Interview quotations: discrepancies between quantitative and qualitative results
	DHI T (*P*, *F*, *E*) HADS-NL EQ-5D-5L	Descriptions: physical	Descriptions: cognitive	Descriptions: emotions	
Patient 1	DHI 62 (24, 20, 16) *A* = 7, *D* = 4 EQ-5D-5L = 0,743	Imbalance, oscillopsia, visual vertigo, no fast head movements	Difficulties with dual tasking	Sadness (suffering, sadness, shame, neglect), fear (nervousness), anger (exasperation, rage)	“brought down”, “life is harder”, “intensely miserable”, “feeling down”, “sad”, “fear of falling”, “feeling unsafe”, “anger”
Patient 2	DHI 74 (20, 26, 28) *A* = 11, *D* = 15 EQ-5D-5L = 0.769	Imbalance, oscillopsia, visual vertigo, reading difficulties, no fast head movements	Difficulties with dual tasking, problems with spatial orientation, concentration problems	Sadness (suffering, sadness, disappointment, neglect), fear (nervousness)	“major limitation”, “I no longer look forward to anything, as it is always disappointing”, “feeling down”, “dark thoughts”, very sad, disappointed in people, fear of falling (during cycling)
Patient 3	DHI 80 (26, 30, 24) *A* = 7, *D* = 7 EQ-5D-5L = 0.439	Imbalance, neck pain	Difficulties with dual tasking, forgetfulness	Anger (irritation, rage)	“quickly irritated”, “aggressive”
Patient 4	DHI 40 (16, 20,4) *A* = 1, *D* = 2 EQ-5D-5L = 0.798	Imbalance, oscillopsia, neck pain	Difficulties with dual tasking	Sadness (shame, neglect), joy (optimism)	“drunk man”, “no balance”, “toddling”, “I need to hold on”(need of support), cycling: “just as if you are being trapped”, moving horizon, “I’m the guilty one”, “vulnerable”, “I adjusted my life accordingly. It has not put me in a situation where I feel run down now or I think this is the beginning of the end”
Patient 5	DHI 46 (16, 20, 10) *A* = 6, *D* = 4 EQ-5D-5L = 0.622	Imbalance, oscillopsia, visual vertigo, tiredness, nausea, headache	Difficulties with dual tasking, concentration problems	Sadness (sadness), fear (nervousness)	“difficult to walk in a straight line”, cycling: “I fall more easily”, swimming: “I don't like it, the water is moving and everything. Can't focus. And I also find it very scary when I go underwater I just don't know what is above and below”, “I just can't keep my head still”, problems with sun shining through trees and oncoming traffic: “missing a peace”, “sometimes tired of it”,
Patient 6	DHI 38 (10, 16, 12) *A* = 7, *D* = 3 EQ-5D-5L = 0.799	Imbalance, oscillopsia, vertigo attacks, tiredness, tinnitus	Difficulties with dual tasking, problems with spatial orientation, concentration problems	Sadness (sadness, neglect), fear (nervousness), anger (irritation)	“wandering”, “drunk”,”I am a fanatic bird watcher … first I have to stop and by then the bird has often flown”(bird watching not possible anymore), “flickering image”, “visual trailing”, vertigo during concentrating for a long time, cycling and oncoming traffic: “I have no control over what comes towards me. if he swerves, then I'm lost because I don't know what to do”, “reacting more sensitively”, “more insecure”, “fear of your instability” (falling), “stress”
Patient 7	DHI 32 (12, 10, 10) *A* = 4, *D* = 3 EQ-5D-5L = 0.805	Imbalance, oscillopsia, no fast head movements, tiredness, tinnitus, lightheadedness	Forgetfulness	Sadness (sadness, shame), fear (nervousness), joy (optimism)	“staggering”, “drunk”, “You’re not able to cycle in a straight line, than you will fall”, stairs and standing up: “need to hold on”, not able to recognize faces or signs during walking, moving horizon, “fast head movements are difficult”, “tiredness”, standing up quickly: “lightheaded”, “forgetfulness”, “moments of feeling down”, “It’s all my fault”, “fear”, fear of falling, fear of walking stairs, “hyperventilating”, “stress”, Wife: “that makes him very sad”
Patient 8	DHI 52 (20, 20, 12) *A* = 10, *D* = 5 EQ-5D-5L = 0.580	Imbalance, oscillopsia, visual vertigo, reading difficulties, no fast head movements, tiredness, lightheadedness	Problems with spatial orientation, concentration problems	Sadness (sadness, shame, neglect), fear (nervousness)	“burnout”, “emotional”, “sadness”, “shame”, “sometimes, I just find it bothersome to be reliant on others” (stopped using antidepressant one week ago)
Patient 9	DHI 64 (22, 26, 16) *A* = 16, *D* = 13 EQ-5D-5L = 0.490	Imbalance, oscillopsia, visual vertigo, reading difficulties, no fast head movements, vertigo attacks, tiredness, tinnitus, neck pain, nausea, lightheadedness, headache	Difficulties with dual tasking, problems with spatial orientation, forgetfulness	Sadness (sadness, shame, neglect), fear (horror), anger (irritation, exasperation, rage)	
Patient 10	DHI 76 (24, 28, 24) *A* = 12, *D* = 12 EQ-5D-5L = 0.625	Imbalance, oscillopsia, visual vertigo, reading difficulties, no fast head movements, tiredness, tinnitus, lightheadedness	Difficulties with dual tasking, concentration problems, forgetfulness	Sadness (suffering, sadness, neglect), fear (horror, nervousness), anger (irritation), joy (optimism)	
Patient 11	DHI 58 (20, 22, 16) *A* = 7, *D* = 9 EQ-5D-5L = 0.498	Imbalance, oscillopsia, visual vertigo, no fast head movements, vertigo attacks, tiredness, nausea, headache, sleeping problems	Difficulties with dual tasking, problems with spatial orientation, concentration problems, forgetfulness	Sadness (suffering, sadness, neglect), fear (horror, nervousness), anger (irritation, exasperation), joy (hope, optimism)	
Patient 12	DHI 56 (20, 24, 12) *A* = 9, *D* = 1 EQ-5D-5L = 0.700	Imbalance, oscillopsia, visual vertigo, reading difficulties, no fast head movements, vertigo attacks, tiredness, tinnitus, nausea, headache	Difficulties with dual tasking, problems with spatial orientation	Sadness (neglect), anger (exasperation), joy (optimism)	“loss of freedom”, “dependent”, “powerlessness up to annoyance”, “insecure”, “incomprehension of others”
Patient 13	DHI 42 (16, 14, 12) *A* = 4, *D* = 3 EQ-5D-5L = 0.837	Imbalance, oscillopsia, visual vertigo		Fear (nervousness), anger (irritation)	“anxiety”, “irritation”
Patient 14	DHI 68 (24, 24, 20) *A* = 2, *D* = 1 EQ-5D-5L = 0.730	Imbalance, oscillopsia, visual vertigo, reading difficulties, no fast head movements, tiredness, tinnitus, restless mind, sleeping problems	Difficulties with dual tasking	Sadness (suffering, disappointment, neglect), fear (nervousness)	“it controls my whole life”, “often, I find it such a pity”, “displeasure”, “If I go away, I won’t go by myself”, “fear of falling”
Patient 15	DHI 62 (20, 22, 20) *A* = 14, *D* = 8 EQ-5D-5L = 0.776	Imbalance, oscillopsia, visual vertigo, reading difficulties, vertigo attacks, tiredness, headache	Difficulties with dual tasking, problems with spatial orientation, forgetfulness	Sadness (neglect), fear (nervousness), anger (irritation)	“dependent”, “more anxious”, “annoying”
Patient 16	DHI 64 (20, 22, 22) *A* = 15, *D* = 14 EQ-5D-5L = 0.589	Imbalance, oscillopsia, visual vertigo, no fast head movements, vertigo attacks, tiredness, tinnitus, neck pain, light-headedness, headache, restless mind	Difficulties with dual tasking, problems with spatial orientation	Sadness (sadness, disappointment, shame, neglect), fear (nervousness)	
Patient 17	DHI 58 (20, 22, 16) *A* = 6, *D* = 7 EQ-5D-5L = 0.608	Imbalance, oscillopsia, visual vertigo, reading difficulties, no fast head movements, tiredness, headache, restless mind	Difficulties with dual tasking, problems with spatial orientation, concentration problems	Sadness (sadness, shame, neglect, sympathy), fear (nervousness), anger (irritation), joy (hope, optimism)	“it spoils everything”, “I feel really guilty”, “you don't want everyone to see it”(embarrassed), “I have to hand it over”, “feeling left out”, “I don't want to appear pathetic”, cycling: “stressful”, “annoying”, “short fuse”, “I try to get everything out of it”
Patient 18	DHI 12 (0, 6, 6) *A* = 0, *D* = 1 EQ-5D-5L = 0.824	Imbalance, oscillopsia, visual vertigo, no fast head movements	Difficulties with dual tasking	Fear (nervousness), love (affection)	“I sway”, “staggering”, “drunk”, “falling”, “fell with the bicycle”, horizon: “moves during walking”, not able to recognize faces or read signs or boxes in the supermarket during walking, “not able to focus”, “hard to look back” (literally: turning head), “fear of losing balance”, “more anxious”, “It takes more effort and more concentration”

The second column presents the results of the quantitative data per patient. “*T* (*P*, *F*, *E*)” represent the total (*T*), physical (*P*), functional (*F*), and emotional (*E*) scores of the DHI questionnaire. “*A*” and “*D*” indicate the anxiety (*A*) and depression (*D*) subscales of the HADS questionnaire. The third–fifth columns show the symptoms described during the semi-structured interviews. The last column demonstrates relevant quotations per patient, which illustrate discrepancies between the quantitative and qualitative results

## Discussion

This qualitative study demonstrated that BV patients suffer from a broad spectrum of symptoms, which can be categorized into physical, cognitive, and emotional domains. These symptoms are provoked and worsened by darkness, uneven ground, triggers, and other specific situations, like driving a car, cycling, being in a supermarket, participating in social activities, etc. These symptoms result in an altered behavior regarding activities. Activities need to be performed slower, with greater attention, or they are avoided. Moreover, these symptoms reduce quality of life. It was illustrated that the current vestibular and quality of life-related questionnaires did not fully capture the complete spectrum of symptoms and burden of disease, experienced by BV patients.

### Symptoms

Imbalance and oscillopsia were the most commonly reported symptoms. This is congruent with previous literature [8, 9]. However, since these symptoms are part of the diagnostic criteria for BV [6], the found percentages could (partially) reflect a possible selection bias. Regarding other symptoms, vertigo attacks occurred in 33% of the interviewed patients, which is in line with previous reports [5, 8]. Neck pain was reported in 22% of the patients, but this might not be fully related to BV itself, since this can occur in patients who suffer from dizziness of a vestibular and non-vestibular origin [43]. The complaints of tinnitus, nausea, and headache symptoms might rather be (partially) related to the etiology that caused BV, than BV itself [2]. Spatial orientation problems described in this study were congruent with previous literature and could (partially) be linked to hippocampal atrophy which occurs as a result of BV [13, 44, 45]. It can be concluded that this prospective study confirmed the results presented in a previous retrospective systematic review: the spectrum of BV symptoms is wider than imbalance and oscillopsia [8].

A certain degree of the symptoms reported could be not specific to BV and might also be related to having a chronic illness. After all, it has been shown that chronic illness can cause higher psychological distress and a higher need of psychosocial support, which could partially worsen the negative emotions and part of the cognitive symptoms, like concentration problems and forgetfulness [46, 47]. However, BV also causes changes in the connectivity of cortical and subcortical structures. This can also influence bodily self-consciousness, emotions and cognition [48, 49]. A multifactorial origin of these symptoms seems, therefore, most likely.

### Behavior

The alteration in behavior seems to correspond with the function of the peripheral vestibular system. The specific contribution of the vestibular system to the whole balance system mainly involves adding speed and automatism to activities, by eliciting very fast reflexes (e.g., vestibulo-ocular reflex, vestibulo-collic reflex, and vestibulo-spinal reflex) that outperform consciously activated mechanisms. These very fast corrections cannot be replaced by other sensory systems like the visual or somatosensory system [50]. Therefore, BV patients need to slow down many of their activities, perform them with more attention, or avoid them. Nevertheless, this does not imply that activities are always performed more slowly and with enhanced attention, or avoided. A single aspect can also be present. For example, BV patients often show a “preferred” walking speed, which is not necessarily slower than their walking speed before BV occurred. After all, a higher walking speed can increase stability due to automatic locomotor patterns by spinal cord mechanisms. However, BV patients still report paying more attention to walking, since they lack the fast vestibular mediated reflexes that can prevent them from falling (increasing risk for falls), e.g., when walking on uneven ground or when tripping [51–54]. This demonstrates that dual tasking can be impaired.

### Comparison of qualitative and quantitative findings

This study chose to collect qualitative data and quantitative data, since these data types are complementary. Qualitative data provide more detailed information about complex situations and more depth of understanding [39, 55–57], while quantitative data provide measurable evidence in known phenomena and the possibility of statistical generalization [39, 56, 57]. The mixed-method approach combines the strengths of both data types, by merging and comparing the non-numerical and numerical data [39].

Discrepancies were found in results between the qualitative (semi-structured interviews) and quantitative (questionnaires) methods. Qualitative data included many items that would indicate a severe disease burden in most of the patients, but some lower individual scores on the DHI and the mean HADS score did not always confirm this. These discrepancies might be explained by a phenomenon called “adaptation”: as a result of the chronically changed situation, patients might change their view of what they consider as normal [58]. These questionnaires are probably less able to capture adaptation than semi-structured interviews in BV [59]. It could also be hypothesized that qualitative data collection is more thorough, because the opportunity exists to ask more questions to get an explanation about the answers and therefore, it is less sensitive to interpretation of the questions. Furthermore, emotions and psychological distress experienced by BV patients are broader than captured with the DHI and HADS. Taking these factors into account, it might be hypothesized that questionnaires like the DHI and HADS do not give an accurate view of symptoms and their consequences in (at least) BV patients [32]. Therefore, it is important to take this into consideration during the development of candidacy criteria and outcome measures for therapeutic interventions such as the vestibular implant, balance belt, noise galvanic vestibular stimulation and rehabilitation [4, 19–24, 60–63]. It should be considered to develop a BV-specific patient-reported outcome measure (PROM) [27, 64] which takes all the relevant symptoms and adaptation to symptoms into account.

### Limitations

These interviews were part of an extensive test day with multiple vestibular tests. The patients who were not able to stop using antidepressants/vestibulosuppressants one week before the interview were excluded from this study. Therefore, the tested patient population might suffer from a possible selection bias (including relatively “fit” BV patients), which may have resulted in an underestimation of severity and frequency of BV symptoms. Furthermore, this study could not determine whether presence and/or severity of symptoms might (partially) be related to age instead of BV, due to the lack of an age-matched control group. However, during the interviews, it was continuously checked whether reported symptoms were related to (the onset of) BV, according to the patients. This approach decreased the chance of including age-related symptoms, although it cannot be excluded that age to a certain extent aggravated the existing BV symptoms.

## Conclusion

This study shows that bilateral vestibulopathy results in a broad spectrum of symptoms in three main domains: physical, cognitive, and emotional. These symptoms illustrate the loss of speed and automatism in the vestibular system, resulting in altered behavior when performing activities: BV patients need to slow down their activities, perform them with more attention, or avoid them. It is demonstrated that the spectrum of symptoms related to BV is much broader than currently addressed by available questionnaires and literature. It might, therefore, be favorable to develop specific candidacy criteria and/or outcome measures for therapeutic interventions, such as the vestibular implant.

## Electronic supplementary material

Below is the link to the electronic supplementary material.

Supplementary file1 (PDF 201 kb)
