# Supplementary material for: Bilateral vestibulopathy: beyond imbalance and oscillopsia
Source: J Neurol. 2020 Oct 8;267(Suppl 1):241–55. doi: 10.1007/s00415-020-10243-5 (PMC7718190; doi:10.1007/s00415-020-10243-5)
Supplement: Supplementary file 1 — Supplementary file1 (PDF 201 kb) [file 415_2020_10243_MOESM1_ESM.pdf]

Supplemental materials

Table 1. Bilateral vestibulopathy symptoms: Physical, Cognitive, Emotions

	First 18 patients (%)	
<u>**Physical**</u>		
- **Imbalance**	**100%**	**18/18**
o Unsteadiness during walking or standing	100%	18/18
o Falling	44%	8/18
o Losing balance during fast movements	61%	11/18
o Keeping balance while cycling	89%	16/18
o Need of support/reference	89%	16/18
o Unsteadiness during other movements	67%	12/18
- **Vision**	**94%**	**17/18**
o Oscillopsia	94%	17/18
o Visual vertigo	78%	14/18
o Reading difficulties	44%	8/18
- **No fast head movements**	**67%**	**12/18**
- **Vertigo attacks**	**33%**	**6/18**
- **Lightheadedness**	**28%**	**5/18**
- **Tinnitus**	**39%**	**7/18**
- **Tiredness**	**67%**	**12/18**
- **Neck pain**	**22%**	**4/18**
- **Nausea**	**22%**	**4/18**
- **Headache**	**39%**	**7/18**
- **Restless mind**	**17%**	**3/18**
- **Sleeping problems**	**11%**	**2/18**
<u>**Cognitive**</u>		
- **Difficulties with dual tasking**	**83%**	**15/18**
- **Problems with spatial orientation**	**56%**	**10/18**
o Disorientation	33%	6/18
o Misjudging distances	56%	10/18
- **Forgetfulness**	**33%**	**6/18**
- **Concentration problems**	**39%**	**7/18**
<u>**Emotions:**</u>		
- **Sadness**	**83%**	**15/18**
o Suffering	28%	5/18
▪ Suffering		
o Sadness	61%	11/18
▪ Depression, despair, hopelessness, gloom, sadness, unhappiness		
o Disappointment	17%	3/18
▪ Disappointment, displeasure		
o Shame	39%	7/18
▪ Guilt, shame		
o Neglect	72%	13/18
▪ Alienation, isolation, neglect, loneliness, rejection, defeat, insecurity, embarrassment.		
o Sympathy	6%	1/18
▪ Pity		
- **Fear**	**83%**	**15/18**
o Horror	17%	3/18
▪ Panic		
o Nervousness	78%	14/18
▪ Anxiety, dread, tenseness, worry, uneasiness.		

- **Anger**	**50%**	**9/18**
o Irritation	44%	8/18
▪ Irritation		
o Exasperation	22%	4/18
▪ Frustration		
o Rage	17%	3/18
▪ Anger, rage		
- **Love**	**6%**	**1/18**
o Affection	6%	1/18
▪ Fondness		
- **Joy**	**11%**	**2/18**
o Contentment	6%	1/18
o Optimism	33%	6/18
▪ Hope, optimism		
- **Surprise**	**0%**	**0/18**

Parrot's Classification of Emotions (2001)

Primary emotion	Secondary emotion	Tertiary emotion
**Love**	Affection	Adoration, affection, love, fondness, liking, attraction, caring, tenderness, compassion, sentimentality
	Lust	Arousal, desire, lust, passion, infatuation
	Longing	Longing
**Joy**	Cheerfulness	Amusement, bliss, cheerfulness, gaiety, glee, jolliness, joviality, joy, delight, enjoyment, gladness, happiness, jubilation, elation, satisfaction, ecstasy, euphoria
	Zest	Enthusiasm, zeal, zest, excitement, thrill, exhilaration
	Contentment	Contentment, pleasure
	Pride	Pride, triumph
	Optimism	Eagerness, hope, optimism
	Enthrallment	Enthrallment, rapture
	Relief	Relief
**Surprise**	Surprise	Amazement, surprise, astonishment
**Anger**	Irritation	Aggravation, irritation, agitation, annoyance, grouchiness, grumpiness
	Exasperation	Exasperation, frustration
	Rage	Anger, rage, outrage, fury, wrath, hostility, ferocity, bitterness, hate, loathing, scorn, spite, vengefulness, dislike, resentment
	Disgust	Disgust, revulsion, contempt
	Envy	Envy, jealousy
	Torment	Torment
**Sadness**	Suffering	Agony, suffering, hurt, anguish
	Sadness	Depression, despair, hopelessness, gloom, glumness, sadness, unhappiness, grief, sorrow, woe, misery, melancholy
	Disappointment	Dismay, disappointment, displeasure
	Shame	Guilt, shame, regret, remorse
	Neglect	Alienation, isolation, neglect, loneliness, rejection, homesickness, defeat, dejection, insecurity, embarrassment, humiliation, insult
	Sympathy	Pity, sympathy
**Fear**	Horror	Alarm, shock, fear, fright, horror, terror, panic, hysteria, mortification
	Nervousness	Anxiety, nervousness, tenseness, uneasiness, apprehension, worry, distress, dread
